# Spontaneous pancreatic undifferentiated pleomorphic sarcoma in a laboratory rat: A case report

**DOI:** 10.1002/ame2.12078

**Published:** 2019-08-26

**Authors:** Petros Ypsilantis, Soultana Meditskou, Maria Lambropoulou, Theodora Papamitsou, Constantinos Simopoulos

**Affiliations:** ^1^ Laboratory of Experimental Surgery and Surgical Research School of Medicine Democritus University of Thrace Alexandroupolis Greece; ^2^ Laboratory of Histology and Embryology Aristotle University of Thessaloniki Thessaloniki Greece; ^3^ Laboratory of Histology and Embryology School of Medicine Democritus University of Thrace Alexandroupolis Greece

**Keywords:** malignant fibrous histiocytoma, pancreas, pleomorphic, rat, undifferentiated sarcoma

## Abstract

We present a case of spontaneous undifferentiated/unclassified sarcoma, of a pleomorphic subtype formerly known as malignant fibrous histiocytoma (UPS/MFH), arising from the pancreas of a laboratory rat. The mass was excised after laparotomy from a 6‐month‐old female laboratory Wistar rat. It presented a giant multilobulated mass of irregular shape, which had arisen from the pancreas and occupied almost the entire peritoneal cavity. Histologically the tumor was characterized by a highly variable morphological pattern, with frequent transitions from storiform to pleomorphic areas. An extensive immunohistochemical examination revealed no specific lines of differentiation. Immunohistochemical positivity was observed only to MIB‐1 (high Ki‐67 proliferation index), vimentin and CD68 antibodies. The diagnosis was compatible with UPS/MFH. To the best of our knowledge, the present case is the first report of a spontaneous primary UPS/MFH arising from the pancreas of a laboratory rat.

## 1 INTRODUCTION

According to a previous World Health Organization (WHO) classification of soft tissue tumors,^1^ malignant fibrous histiocytomas (MFH) were a group of tumors with histological cytocharacteristics resembling histiocytes and fibroblasts and were considered to be synonymous with undifferentiated pleomorphic sarcomas.^1^, ^2^ According to the latest WHO classification of soft tissue tumors,^3^ UPS/MFH is a subtype of the new major category “undifferentiated/unclassified soft tissue sarcomas”, a term which is now reserved for sarcomas that lack specific lines of differentiation.^3^, ^4^, ^5^ The subtypes of undifferentiated/unclassified soft tissue sarcomas include (a) undifferentiated spindle cell sarcomas, (b) undifferentiated round cell sarcomas, (c) undifferentiated epithelioid sarcomas, (d) undifferentiated pleomorphic sarcomas, and (e) undifferentiated sarcomas not otherwise specified.^3^, ^4^

The sarcomas previously diagnosed as MFH are the most common type of soft tissue sarcoma in human adults.^6^ They mostly occur in the deep soft tissues of the extremities, but may also develop in the trunk, urogenital track, abdominal cavity, and retroperitoneum. The tumor often grows rapidly and becomes quite large.^6^ There are only rare cases of UPS/MFH arising from the pancreas in humans.^7^, ^8^, ^9^, ^10^, ^11^, ^12^, ^13^

In the laboratory rat, although UPS/MFH can be chemically induced,^14^, ^15^, ^16^, ^17^ it only very rarely occurs spontaneously.^18^, ^19^ To the authors' knowledge, this is the first report of a spontaneous UPS/MFH arising from the pancreas of a laboratory rat.

## 2 MATERIALS AND METHODS

A female Wistar rat, 6 months of age, weighing 290 g, with a 20 cm crown‐rump length, from the rat colony of our laboratory was assigned to be subjected to laparotomy under the terms of a surgical experimental protocol. It was group‐housed in a Makrolon cage, with two other rats, at 20‐22°C room temperature, on a 12‐hour light/12‐hour dark cycle and was provided with commercial pelleted diet and tap water ad libitum. The facilities and experiments were in accordance with Directive 86/609/EEC for the care and use of laboratory animals.

**Figure 1:**
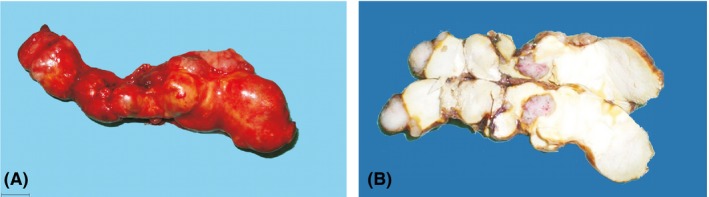
A, Gross appearance of the mass. B, Gross section of the mass following formalin fixation. The cut surface of the mass is irregular, solid, yellowish‐white with red and white areas. Bar: 1 cm

After midline laparotomy, a tumor arising from the pancreas was revealed and excised. This was a solid encapsulated multilobular mass of irregular shape (Figure 1). No tumor lesion to other organs was noted. The animal was then euthanized by exsanguination.

Macroscopically, the excised mass weighed 26 g, was 10.1 cm in length and 3.5 cm at its maximum width. The cut surface of the mass was solid, of a yellowish‐white color with dark reddish and white areas (Figure 1B).

The mass was fixed in 10% phosphate‐buffered formalin and embedded in paraffin according to standard procedures. Histopathological examination was performed on 4 µm hematoxylin‐eosin (H&E) stained sections. In addition, two‐step immunohistochemical staining was performed using the biotin complex EnVision + system (Dako Cytomation). Antibodies against the following antigens were used: vimentin, CD‐68, desmin, SMA, S100 protein, EMA, cytokeratin AE1/AE3, CD117, MIB‐1(Ki‐67), and CD34 (Dako Corporation). Finally, bound antibody complexes were stained for 10 minutes with 0.05% diaminobenzidine. The histochemical and immunohistochemical stained sections were examined under a Nikon Eclipse 50i microscope.

## 3 RESULTS

**Figure 2:**
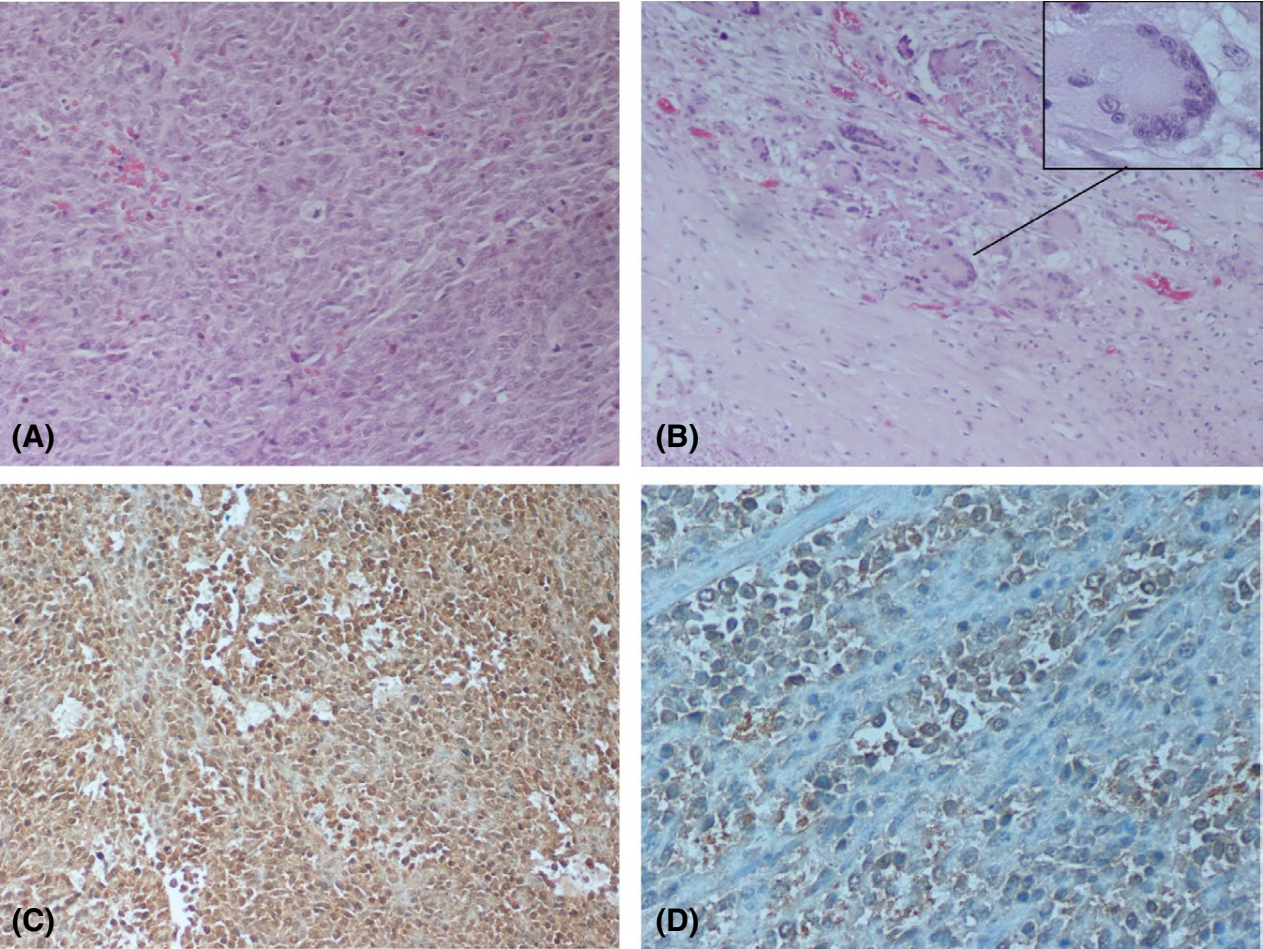
Representative microphotographs of the H&E and immunostained tumor. A, Pleomorphic areas containing plump fibroblastic cells and rounded histiocytic‐like cells (H&E × 200). B, Multinucleated cells with bizarre nuclei (inset) (H&E × 100). C, Positive immunostaining to vimentin (×200). D, Positive immunostaining to CD68 (×400)

Histopathological examination revealed a highly variable morphological pattern with frequent transitions from storiform to pleomorphic areas and scattered areas of necrosis. The lesions mainly consisted of plump spindle cells arranged in short fascicles in a cartwheel or storiform pattern around slit‐like vessels and pleomorphic areas with plump fibroblastic‐like cells and rounded histiocytic‐like cells arranged haphazardly, without any particular orientation with regard to vessels (Figure 2A). Pleomorphic mono‐ or multinucleated cells with bizarre nuclei (Touton‐like cells) were intermingled in the lesion (Figure 2B). There were histiocyte‐like cells containing cellular debris in the cytoplasm. Chronic inflammatory cells (lymphocytes and plasma cells) were scattered throughout the tumour with a predilection to the periphery of the lesion and the perivascular spaces. Immunohistochemistry revealed a high proliferation rate with a 20% Ki‐67 labeling index. The tumour cells were positive to vimentin (Figure 2C) and CD68 (Figure 2D), and negative to desmin, SMA, S100 protein, EMA, cytokeratins AE1/AE3, CD117, and CD34 antibodies.

According to the current WHO classification, the diagnosis was compatible with a high‐grade undifferentiated/unclassified sarcoma of pleomorphic subtype.

## 4 DISCUSSION

Primary UPS/MFHs are rarely localized to the pancreas, with less than 25 cases of previously termed MFHs or UPS/MFHs being reported in humans to date.^7^, ^8^, ^9^, ^10^, ^11^, ^12^, ^13^ UPS/MFH is a soft tissue sarcoma mostly occurring in middle and late adulthood. Although UPS/MFH has also been described in children,^6^, ^20^, ^21^ localization in the pancreas has never been reported at this age. Three histological types of pancreatic sarcoma previously diagnosed as MFH have been observed; storiform/pleomorphic, myxoid, and giant cell.^9^ Our case fitted the storiform/pleomorphic type, being characterized by the presence of highly pleomorphic tumor cells and a storiform pattern of growth.^6^

The only spontaneous pancreatic sarcomas recorded to date in the rat have been a hemangiosarcoma^22^ and a histiocytic sarcoma,^23^ while spontaneous MFHs have been described only in the subcutaneous tissue of a 24‐month‐old rat^19^ and the left thoracic region of an 18‐month‐old rat.^18^

In the present case, a UPS/MFH tumour, arising from the pancreas and occupying almost the entire peritoneal cavity, was revealed in a 6‐month‐old female rat. This was an exceptionally large mass (10.1 cm in length) compared to human pancreatic UPS/MFHs, whose largest recorded dimension is 35 cm.^9^ In the light of the high Ki‐67 labeling index, obtained immunohistochemically, the surprisingly large tumor found in our young adult rat provides evidence of the aggressive nature of the malignancy. Furthermore, taking into account a report that in an experimentally induced MFH it took 120 days after intramuscular benzopyrene injection for overt MFH to develop,^16^ we can assume that the tumor may have begun to develop a few months earlier. In the experimental setting, other methods of chemical induction of MFH in the rat include the subcutaneous administration of 4‐hydroxyaminoquinoline‐1‐oxide^14^, ^17^ or 7,12‐dimethylbenzanthracene^24^ and the intra‐articular injection of methylcholanthrene.^15^ In this case report, there was no administration of or exposure to any toxic substance to justify the early onset of the tumor development.

In our case study, immunohistochemical analysis revealed no specific lines of differentiation. Immunohistochemical positivity for CD68 (a histiocytic marker also highly expressed in cells of monocyte lineage and circulating and tissue macrophages) is frequently reported in undifferentiated sarcomas, and this was observed in the present case. It therefore seems that this demonstrates the existence of high numbers of tumor infiltrating histiocytes rather than true histiocytic differentiation of the neoplastic cells.^5^

The size of the tumor and the depth of its location are considered the two most important prognostic factors in evaluating the capacity to metastasize of tumors formerly known as MFHs.^6^ The prognosis of pancreatic MFH is usually poor for long‐ term survival.^9^ In our case, despite the large size of the tumor and the involvement of the pancreas, no metastasis was noted.

## 5 CONCLUSIONS

The present case is the first report of a spontaneous primary undifferentiated/unclassified soft tissue sarcoma of the pleomorphic subtype arising from the pancreas of a laboratory rat. The tumor was an exceptionally large pancreatic UPS/MFH. Based on the age of the rat, we can assume that this type of malignancy may develop in early adulthood or even in childhood.

## CONFLICT OF INTEREST

None.

## AUTHOR CONTRIBUTIONS

PY performed the laparotomy and mass excision, carried out data management, participated in designing the study, researching the background literature and drafting of the manuscript. ML, SM, and TP performed histopathology, histochemical and immunohistochemical examinations, evaluated the results, researched the background literature and drafted the manuscript. CS participated in the coordination and drafting of the manuscript. All the authors read and approved the final manuscript.
